# Efficacy of fipronil combined with permethrin commercial spot on (Effitix®) preventing *Culex pipiens* from feeding on dogs

**DOI:** 10.1007/s00436-015-4397-z

**Published:** 2015-03-05

**Authors:** Michel Franc, Emmanuel Lienard, Philippe Jacquiet, Stephane Bonneau, Emilie Bouhsira

**Affiliations:** 1 INP Ecole Nationale Vétérinaire, 23 chemin des capelles, 31076 Toulouse, Cedex France; 2 R&D, Virbac A.H. 1re avenue 2065 M-LID, 06511 Carros, France

**Keywords:** *Culex pipiens*, Permethrin, Fipronil, Antifeeding-effect, Dogs, Effitix®

## Abstract

A controlled clinical trial was carried out to assess the adulticidal and anti-feeding effectiveness of a spot-on combining fipronil and permethrin (Effitix®, Virbac, Carros, France) in preventing *Culex pipiens* from feeding on dogs. Twelve dogs with equal sensitivity to mosquitoes were included in the study and divided into two groups of six dogs: an untreated control group and a group treated with Effitix®. All dogs were challenged with 80 females *C. pipiens* for 90 ± 5 min on days −7, 1, 7, 14, 21, and 28 (day 0 being treatment day). The number of engorged, dead, and live mosquitoes was determined after each exposure to treated and untreated dogs. Dead mosquitoes were also counted 24 h after exposure. The anti-feeding effect of the spot-on formulation was 100, 99.5, 97.7, 98.3, and 96.7 % on days 1, 7, 14, 21, and 28, respectively. The mortality effect was 66.6, 55.9, 38, 17.2, and 12.3 % on days 1, 7, 14, 21, and 28, respectively. At each challenge point, the mortality and anti-feeding effects on mosquitoes were significantly different between the control and treated group (*p* < 0.05). The results indicate that a combination of permethrin and fipronil could be used as an effective mosquito control strategy in dogs and is therefore recommended for use in a dirofilariasis prevention program.

## Introduction

The two species of mosquitoes, *Culex pipiens* and *Aedes albopictus*, are important vectors of *Dirofilaria immitis* and *Dirofilaria repens* worldwide (Licitra et al. 2010; McKay et al. 2013). *D. immitis*, the agent of heartworm disease, causes severe disorders and even death in dogs in many parts of the world (McCall et al. 2008). The prevalence of both dirofilariasis is very high in the USA (Carleton and Tolbert 2004; Bowman et al. 2007; McKay et al. 2013), in Central America (Bolio-Gonzalez et al. 2007; Caro-Gonzalez et al. 2011), Asia (Oi et al. 2014), Russia (Ermakova et al. 2014), and in some European countries (Genchi et al. 2011). Endemic areas are present in the south of France, in Italy (Capelli et al. 2013; Giangaspero et al. 2013), Spain (Montoya-Alonso et al. 2010), Portugal (Santa-Ana et al. 2006), Germany (Sassnau et al. 2014), Poland (Demiaszkiewicz et al. 2014), Hungary (Farkas et al. 2014; Tolnai et al. 2014), and in Romania (Mircean et al. 2012). In addition, the filarial nematodes, *D. immitis* and *D. repens*, are zoonotic agents (Theis, 2005). In humans, ocular, subcutaneous, and pulmonary forms have been reported (McCall et al. 2008; Kalogeropoulos et al. 2014; Otranto et al 2011a, 2011b).

An integrated control program against dirofilariasis may be implemented by the association of macrocyclic lactones and the application of insecticides with an antifeeding effect on mosquitoes (Hellmann et al. 2011, Snyder et al. 2011; Genchi et al. 2013; Traversa et al. 2013; Di Cesare et al. 2014). Pyrethroids such as permethrin and deltamethrin are known to be effective against sandflies and mosquitoes and are widely used in companion animals (Beugnet and Franc 2012). Other molecules such as fipronil, metaflumizone, and pyriprole are effective against fleas and tick, but could not be used to prevent mosquitoes from biting dogs (Bouhsira et al. 2009).

The aim of the study was to evaluate the adulticidal (or mortality) and repellent effects of a spot-on containing fipronil and permethrin (Effitix®, Virbac, Carros, France) on *C. pipiens* in dogs.

## Materials and methods

The study was conducted in the National Veterinary School of Toulouse (ENVT) and was a single-center, randomized, blinded, controlled efficacy study on two groups of seven dogs each. Dogs were handled in accordance with the Animal Welfare and Good Clinical Practice, and the study protocol was approved by the Ethics Committee of Midi-Pyrenees. All personnel involved in the collection of efficacy data were blinded to the treatment.

### Dogs

Five male and seven female Beagle dogs (3 years of age and weighing between 8.02 and 10.94 kg) were included in the study. They had not been exposed to ectoparasiticides for 3 months prior to the inclusion and remained in good health throughout the study. They were housed in cages individually and had a 4-h daily access to a 2 × 4 m concrete run without contact with another dog. To avoid cross-contamination, treated and untreated dogs were placed in two different exercise areas. Each dog was identified with the number of a subcutaneously implanted microchip. They were fed a commercial dry dog food with a ration that maintained the animal in a healthy physical state. Water was available ad libitum through automatic lickers. Dogs were maintained and handled with due regard for their welfare and were acclimatized to the caged environment for 17 days prior to treatment. They were observed daily for their general health conditions throughout the trial. No concurrent medication was needed to be given during the study.

On day −7, each dog was challenged with 80 unfed adult females of *C. pipiens*. The number of engorged female mosquitoes was used for ranking and group allocation. Dogs were ranked in descending order according to their individual pre-treatment mosquito’s engorgement status. They were then introduced into blocks of two animals each, and within each block, dogs were randomly allocated into two groups: treatment or control group.

### Mosquito maintenance and supply

The *C. pipiens* exposure was performed using laboratory-reared adults (females only). This mosquito strain was obtained from the Interdepartmental Agreement for Mosquito Control (EID) and was maintained at ENVT under laboratory conditions since 2001 using a 3-week egg to adult.

### Treatment

The six dogs from the control group (group A) remained untreated, and the six dogs from the treated group (group B) received on day 0 a spot-on combination of permethrin and fipronil: one pipette of 1.1 ml (593.4 mg of permethrin and 67.7 mg of fipronil) for dogs weighing between 4.1 and 10.0 kg and one pipette of 2.2 ml (137.2 mg of fipronil and 1197.8 mg of permethrin) for dogs weighing between 10.1 and 20 kg. Treatment dosages were within the range of 67.7–137.2 mg kg^−1^ for permethrin and 7–13.5 mg kg^−1^ for fipronil. For all treated animals, the formulation was applied according to the manufacturer’s instructions by parting the hair and applying the formulation directly onto the skin in two areas: between the shoulder blades and the lumbar area. All dogs were observed at 2 and 4 h after treatment for any adverse reaction to the product.

### Experimental procedure

The 12 dogs were infested with 80 (±2) *C. pipiens* for a total of six times. Two days before exposure, mosquitoes were aspirated from their breeding cage with a vacuum pump and then placed in challenge nets (80 ± 2 females per net) with access to water-soaked cotton and honey. The mosquito challenge assessment cages (60 cm × 40 cm × 50 cm) were constructed from mosquito netting mounted on a wooden frame and placed in environmentally controlled rooms. Mosquitoes were fasted 24 h prior to exposure to dogs by removing honey from the cages.

Before exposure, dogs were sedated by intramuscular injection of medetomidine (Dexdomitor®, Pfizer Santé animale, Paris, France), ketamine (Clorketam®, Laboratoire Vetoquinol S.A., Lure, France), and diazepam(Valium®, Roche injectable, Neuilly s/ Seine, France) at a dose rate of 4 μg/kg, 9 mg/kg, and 5 mg/dog, respectively, and then placed in individual infestation proof nets containing mosquitoes. The dosage of the anesthetic was calculated so as to immobilize dogs for 90 min. During infestation, treated dogs and control dogs were placed in separated infestation rooms where temperature and relative humidity were maintained between 25 and 26 °C and between 58 and 72 %, respectively. Cages and nets were thoroughly cleaned after each mosquito challenge.

After 90 ± 5 min of exposure, the dogs were carefully taken out of the net and examined for any dead mosquito on their body and then placed back in their cage. All live mosquitoes were aspirated from each challenge net using a vacuum pump and were categorized as live engorged or non-engorged. All dead mosquitoes were collected, counted, and categorized as dead non-engorged or dead engorged. On days −7, 1, 7, 14, 21, and 28, live mosquitoes recovered from individual animals at the end of exposure were placed in separate nets and kept in the experimental room. The mosquitoes were fed on sugar–water and checked for mortality after 24 h. Then, all remaining mosquitoes were discarded.

## Data analysis

### Antifeeding effect

For each time point after exposure, the antifeeding effect was calculated as described by:

$$ \mathrm{Antifeeding}\;\mathrm{e}\mathrm{ffect}=100*\frac{\mathrm{Ce}-\mathrm{T}\mathrm{e}}{\mathrm{Ce}} $$

where Ce was the arithmetic mean of engorged female mosquitoes (live engorged and dead engorged) for the control group and Te was the arithmetic mean of the engorged female mosquitoes for the treated group.

### Mortality effect

For each time point after exposure, the mortality effect was evaluated for each group as described by:

$$ \mathrm{Mortality}\;\mathrm{effect}=100*\frac{\mathrm{Cl} - \mathrm{Tl}}{\mathrm{Cl}} $$

where Cl was the arithmetic mean of live female mosquitoes (live engorged and live unengorged) for the control group and Tl was the arithmetic mean of the live female mosquitoes for the treated group.

The mortality effect was calculated at 90 min and 24 h post-exposure.

### Statistical analysis

Both groups were compared for the number of engorged females and the number of dead females at each challenge point using the non-parametric test of Kruskal–Wallis. The analyses were performed with Systat 9 software; differences were considered significant at *p* < 0.05.

## Results

No adverse events relative to treatment were reported.

### Antifeeding effect on mosquitoes

**Fig. 1:**
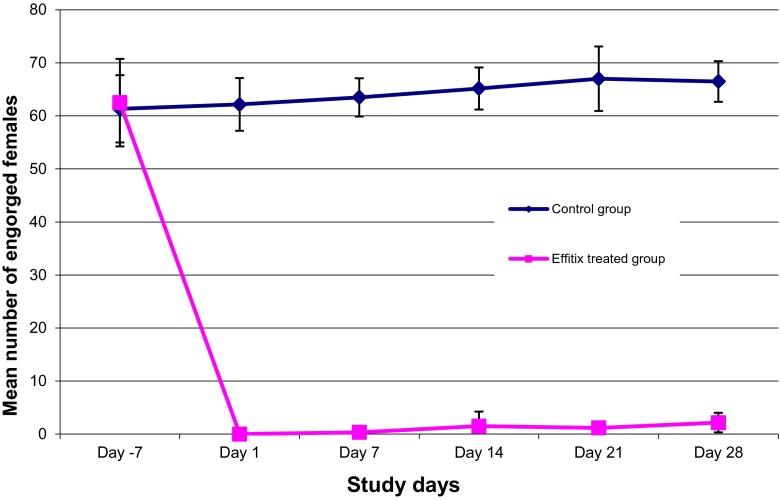
Mean number of engorged *Culex pipiens* females after 1 h exposure to control and treated dogs. Dogs were treated on day 0 with a permethrin and fipronil combination spot-on and then weekly challenged with 80 *Culex pipiens* females

The 12 dogs included in the study demonstrated adequate pre-treatment parasite-holding ability (i.e., over 50 % of engorged females per dog; Fig. 1). On day −7, the percentage of engorged females was 78.1 and 76.6 % for the treated and control group, respectively. All control dogs maintained an adequate number of engorged females (i.e., between 76.6 and 83.7 %) throughout the study (Fig. 1). The treatment had an anti-feeding effect between 100 and 99.5 % during the first 2 weeks and between 98.3 and 96.7 % until the end of the trial (Table 1).

**Table 1:** Mortality and antifeeding effect of a permethrin and fipronil combination against *Culex pipiens* on dogs

		Day 1	Day 7	Day 14	Day 21	Day 28
Mortality effect (%)	1.5 h	66.6	55.9	38	17.2	12.3
	24 h	69.1	58.1	38	17.8	15.1
Anti-feeding effect (%)	1 h	100	99.5	97.7	98.3	96.7

At each challenge point post-treatment, the difference in engorgement status of *C. pipiens* females between treated and controlled group was significant (*p* < 0.05).

### Mortality effect on mosquitoes

The mortality effects of the treatment calculated at 1 and 24 h post-exposure to treated dogs are reported in Table 1. The mortality effect observed at 1 h ranged from 66.6 to 55.9 % in the first 2 weeks and then decreased dramatically to values ranging from 38 to 12.3 % until the end of the study. The mortality effect of the formulation has not increased within the 24 h post-exposure and was close to the one obtained at 1 h. At each challenge point, there was a significant difference (*p* < 0.05) in the number of dead mosquitoes found at 1 and 24 h of exposure between the treated and control group.

## Discussion

The aim of the study was to determine the antifeeding (or repellency) and mortality (or insecticidal) efficacies of a new formulation combining fipronil and permethrin (Effitix®, Virbac, Carros, France) against a European strain of *C. pipiens* in dogs. The formulation was administered according to the manufacturer’s recommendations and the dogs received, according to their weight, between 67.7 and 137.2 mg.kg^−1^of permethrin and between 7 and 13.5 mg.kg^−1^of fipronil. In these conditions, the treatment provided an immediate efficacy of 100 % at 24 h after the administration. Then, the formulation provided an excellent inhibition of feeding which remained above 96.7 % for the 4 weeks of the study.

The repellency of fipronil combined with permethrin obtained in our study was higher than the repellency obtained with the same association against an American strain of *C. pipiens* (Fankhauser et al. 2015). In this study, the authors obtained a repellency rate of 99.4, 98.9, 94.7, 91.7, and 90.4 % on days 1, 7, 14, 21, and 28, respectively. This could be explained by the fact that dogs were treated at the minimal dose of the product, i.e., 50.48 mg kg^−1^ of permethrin and 6.76 mg kg^−1^ of fipronil versus 67.7 mg kg^−1^ of permethrin and 7 mg kg^−1^ of fipronil in our study. A previous study had been carried out in the same laboratory as the current study to assess the repellency and the insecticidal efficacy of fipronil combined with (S) methoprene on the same European strain of *C. pipiens* (Bouhsira et al. 2009). The repellency rate was 40.2 % on day 1 and 45.8 % on day 7 respectively while the insecticidal efficacy was 32.3 and 51.8 % on the same days. This combination had a reduced activity on *C. pipiens* which could not be considered as efficient enough to protect dogs against this mosquito. Therefore, the excellent inhibition of feeding provided by Effitix® is mainly due to permethrin.

The product demonstrated mortality or insecticidal efficacy close to 60 % the first week after treatment which then decreased on days 14, 21, and 28. Fankhauser et al. (2015) obtained an insecticidal efficacy between 92.1 and 26.9 %. This lower insecticidal efficacy was explained by the strong repellent effect on *C. pipiens*, which could prevent them from landing on the treated animals and therefore limiting contact with the insecticide molecules (Fankhauser et al. 2015).

In conclusion, the new fipronil and permethrin ectoparasiticide combination offers a protection against *C. pipiens* in dogs for 1 month following a single topical application. This treatment could contribute to the reduction of stress and annoyance caused by the bite of mosquitoes, and more importantly, it may reduce the risk of heartworm transmission in animals living in or travelling to dirofilariasis endemic areas. However, it should not be seen as a substitute for heartworm prevention treatment but should be part of an integrated prevention program.
